# Ten cases of *Mycobacterium avium* subsp. *hominissuis* infections linked to equine abortions in Japan, 2018–2019

**DOI:** 10.1002/vms3.411

**Published:** 2020-12-18

**Authors:** Yuta Kinoshita, Mari Takechi, Eri Uchida‐Fujii, Kunio Miyazawa, Toshio Nukada, Hidekazu Niwa

**Affiliations:** ^1^ Microbiology Division Equine Research Institute Japan Racing Association Shimotsuke Japan; ^2^ Hokkaido Hidaka Livestock Hygiene Service Center Hidakagunshinhidakacho Japan

**Keywords:** aborted fetus, horses, *Mycobacterium avium* complex, placenta diseases

## Abstract

Bacterial placentitis in horses commonly results in abortion, premature birth or compromised neonatal foal health. Although mycobacterial infections are generally uncommon in horses, 10 equine abortion cases caused by *Mycobacterium avium* subsp. *hominissuis* (MAH) infections occurred between 2018 and 2019 in Japan. They occurred on seven Thoroughbred horse farms in the Hidaka district of Hokkaido, but direct contact among the mares on different farms was not recorded. Most cases were characterized by extensive pathological lesions of the placenta, which are not typical in cases of common pathogenic bacteria such as *Streptococcus zooepidemicus* and *Escherichia coli*. All abortions featured white–yellow exudates on the surface of the placenta. Mycobacterial granuloma formations were histologically found in the placenta and fetal organs, and acid‐fast bacteria were isolated from the placenta, fetal samples (heart, lung, liver, kidney, spleen and stomach contents) or uterine lavage fluid. The greatest number of bacteria was isolated from necrotic lesions on the placenta, which could be an important site for bacterial isolation in mycobacterial equine abortions. The isolates were identified as MAH based on internal genome sequences. In variable number tandem repeat analysis, all patterns of the strains were identical. Single nucleotide polymorphism analysis of the core genome grouped all strains in the II‐a/SC3 subcluster. Both results reveal that these strains share the same genetic background, suggesting that the horses had been infected by the same unknown contagious source.

## Graphical abstract

This article reports 10 equine abortions cases caused by *Mycobacterial avium* subsp. hominissuis infections. We confirmed the isolated strains shared the same genetic background, suggesting that the horses had been infected by the same contagious source.

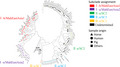

## 1 INTRODUCTION

Equine abortions can lead to tremendous economic losses for horse breeders and are divided into infectious and non‐infectious causes. Infectious abortions are commonly attributed to viruses and bacteria, and fungi can occasionally lead to equine abortions (Giles et al., 1993). Common viral agents that lead fetal deaths are equine herpesvirus (EHV)‐1, EHV‐4 and equine arteritis virus, and common bacterial agents are *Streptococcus zooepidemicus*, *Escherichia coli* and *Pseudomonas aeruginosa* (Bazanow et al., 2014; LeBlanc, 2010). A retrospective cohort study of 2,137 equine abortions placed bacterial infection as the second common infectious cause (7.3%) after viral infection (10.1%) in Thoroughbred horses in Japan (Murase et al., 2017). Of the bacteria, *S. zooepidemicus* and *E. coli* were the most frequently isolated in Japan, which is common with findings from other countries (Murase et al., 2017).

Horses are considered to be highly resistant to mycobacterial infections (Pavlik et al., 2004; Thorel et al., 1997). However, occasional cases are reported. Of the cases investigated, *Mycobacterium avium* complex (MAC) members were the most common pathogens (Pavlik et al., 2004). *Mycobacterium avium* subsp. *hominissuis* (MAH) is a member of the MAC and is frequently isolated from pigs and humans (Mijs et al., 2002). In horses, most MAH infections have taken the form of tubercular lesions in the liver, spleen, lung tissue, colon, lymph nodes and other organs (Pavlik et al., 2004). Five cases of equine abortion associated with fetal mycobacterial infections have been reported: one case of *M. terrae* in Australia in 1981 (Tasler & Hartley, 1981), one case of MAC infection in the USA in 1991 (Cline et al., 1991), one of MAC in Canada in 1996 (Helie & Higgins, 1996), one case of a novel *Mycobacterium* species in 2012 (Johnson et al., 2012) and one case of MAH in Japan in 2014 (Sano et al., 2014). In this paper, we report an outbreak of MAH infection resulting in 10 equine abortions in Japan.

## 2 MATERIALS AND METHODS

### 2.1 Cases of equine abortions

We describe 10 abortions in Thoroughbred horses caused by MAH infections that occurred in 2018 and 2019 (Table 1). The 10 mares (average age ± *SD*, 15.1 ± 4.0 years) were kept on seven horse farms located within a 30‐km radius in the Hidaka district of Hokkaido, Japan. No evidence of direct contact between the mares was found. The fetal ages at the time of the abortions ranged from 148 to 303 days (average age ± *SD*, 222.6 ± 46.1 days). All abortions except case no. 4, from which placental samples were not available, were further pathologically investigated. Equine herpesvirus type 1 was tested in fetal lung and thymus by using a loop‐mediated isothermal amplification method (Nemoto et al., 2010). Uterine lavage was conducted in case no. 4 after the abortion and uterine lavage fluid was sampled for the following bacterial isolation.

**TABLE 1:** Detail information of aborted Thoroughbred horses

Case no.	Date of abortion	Farm	Mare's age (years)	Fetal age (days)	Fetal body weight	Origin and name of MAH strain^a^	Biosample accession number	Note
1	2018/1/27	A	10	238	11 kg	Stomach content, JP‐H‐1	SAMD00184123	Complete genome sequence of MAH strain JP‐H−1 is available under the accession numbers of AP020326 ‐ AP020329
2	2018/2/9	B	18	260	12 kg	Stomach content, JP‐H‐2	SAMD00185689	
3	2018/2/18	C	13	303	22 kg	Fetal lung, JP‐H‐3	SAMD00185690	
4	2018/8/11	D	14	148	Unknown	Uterine lavage fluid, JP‐H‐4	SAMD00185691	
5	2018/12/1	C	19	170	3 kg	Stomach content, JP‐H‐5	SAMD00185692	
6	2018/12/9	E	11	205	7 kg	Stomach content, JP‐H‐6	SAMD00185693	
7	2018/12/12	B	10	267	14 kg	Stomach content, JP‐H‐7	SAMD00185694	
8	2018/12/18	D	17	210	8 kg	Stomach content, JP‐H‐8	SAMD00185695	
9	2018/12/17	F	18	215	11 kg	Stomach content, JP‐H‐9	SAMD00185696	
10	2019/2/13	G	21	210	4.5 kg	Stomach content, JP‐H‐12	SAMD00185698	In addition to MAH, *Streptococcus zooepidemicus* was isolated from placenta and fetal stomach contents.

^a^ *Mycobacterium avium* subsp. *hominissuis* (MAH).

### 2.2 Bacterial isolation

For isolation of acid‐fast bacteria, the placenta, fetal samples (heart, lung, liver, kidney, spleen and stomach contents) or uterine lavage fluid from each case were used. Except for stomach contents, 1 g of each specimen was homogenized in a 15‐ml glass homogenizer. Two volumes of N‐acetyl‐L‐cysteine‐sodium hydroxide (2%) was added to the bacterial suspensions of each specimen (except for stomach contents) and incubated at room temperature for 15 min. Phosphate buffer solution (PBS, pH 6.8) was added up to 15 ml and the mixture was centrifuged at 3,000 *g* for 20 min. The supernatant was discarded and the pellets were resuspended in PBS. Then 100 µl of the mixed suspensions or stomach contents were inoculated on Middlebrook 7H11 agar plates (Kyokuto Pharmaceutical Industrial Co., Ltd.) and incubated at 37°C for 2 weeks in a 5% CO_2_ atmosphere. For standard bacterial isolation, cut surfaces of each organ were directly stamped onto sheep blood agar plates and deoxycholate hydrogen sulphide lactose agar plates, and the plates were incubated at 37°C for 2 days in a 5% CO_2_ atmosphere.

### 2.3 DNA extraction and bacterial identification

The genomic DNA of each MAH strain was obtained for whole‐genome sequencing following a previous method (Bouillaut, McBride, & Sorg, 2011) with the addition of zirconia beads beating step before the proteinase K process. DNA quality was verified on a Nanodrop 2000c spectrophotometer and a Qubit 3 fluorometer (both Thermo Fisher Scientific Inc.). Bacteria were identified by PCR‐based methods to investigate the presence/absence of several internal sequences (Campora et al., 2011; Chae et al., 2017).

### 2.4 Variable number tandem repeat analysis

For variable number tandem repeat (VNTR) analysis, 15 loci of the *M. avium* tandem repeat (MATR)—MATR‐1–9, 11–16—were amplified with the primer sets described in a previous report (Inagaki et al., 2009). All PCR assays were conducted using the EmeraldAmp PCR master mix (Takara Bio Inc.). The reaction mixtures contained 2 μl of genomic DNA, 25 μl of 2 × premix, oligonucleotide primers (0.1 mM) and 4% dimethyl sulfoxide (DMSO) in a final volume of 50 μl. DNA was amplified with an initial denaturation step of 98°C for 3 min; 35 cycles of denaturation at 98°C for 10 s, annealing at 67°C for 30 s, and extension at 72°C for 1 min; and a final extension at 72°C for 3 min. The amplicons were purified by using Nucleospin Gel and PCR Clean‐Up (Takara Bio Inc.). The purified amplicons were electrophoresed in E‐gel EX 2% agarose (Invitrogen Japan KK) and visualized under an E‐gel Safe imager (Invitrogen Japan KK).

### 2.5 Whole‐genome sequencing and SNPs analysis

Whole‐genome sequencing was carried out on an Ion Torrent Personal Genome Machine (PGM) using the Ion 318 Chip v2 BC and Ion PGM Hi‐Q View Chef Reagents (all Thermo Fisher Scientific Inc.). Short reads were trimmed in Sickle v.1.33 software (Joshi & Fass, 2011) using a quality threshold of 25 and a length threshold of 100 bp. Single nucleotide polymorphism (SNP) analysis used 137 sets of MAH genomic information; the 10 MAH strains from this study and 127 registered MAH information sets from the National Center for Biotechnology Information database. Snippy v.4.4.1 software (Seemann, 2015) was used to identify core‐genome SNPs among the 137 genomes, and MEGA X software (Kumar et al., 2018) was used to create a phylogenetic tree based on 60,511 SNPs by the maximum likelihood method. Five subclusters (I‐a/MahEastAsia2, I‐b/MahEastAsia1, II‐a/SC2, II‐a/SC3 and III‐a/SC1) were identified (Uchiya et al., 2017; Yano et al., 2017).

## 3 RESULTS AND DISCUSSION

**FIGURE 1:**
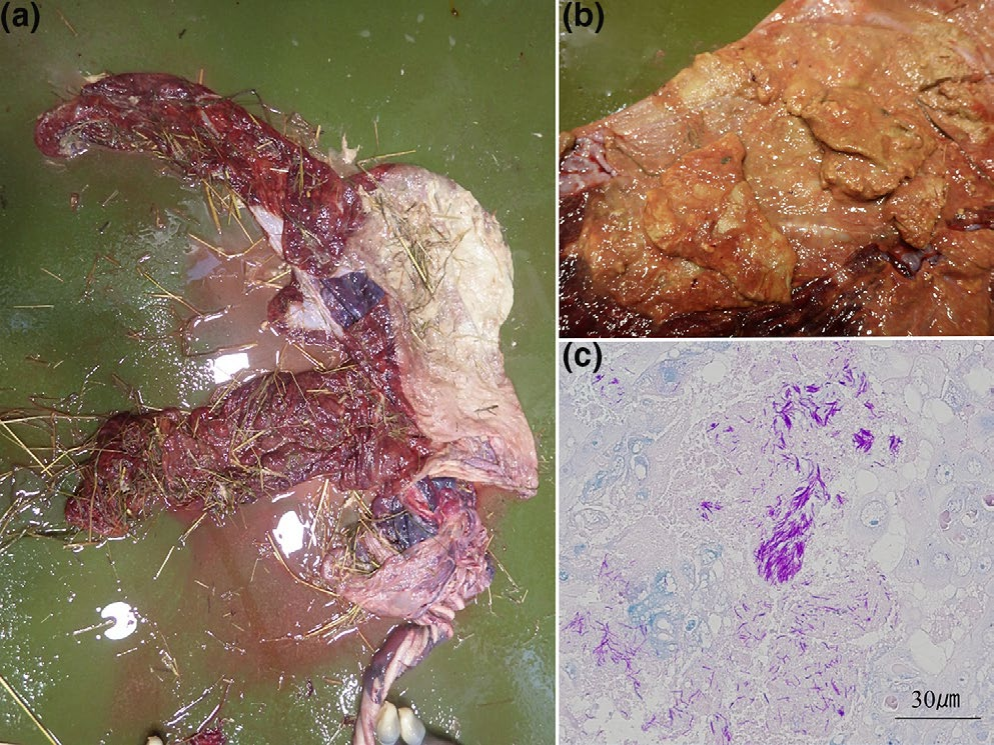
Aborted material and bacterial staining: (a) Extensive placentitis in case no. 1. (b) Yellow–white exudate in case no. 7. (c) Ziehl–Neelsen staining in necrotic lesions of the placenta in case no. 1

Ten cases of infectious placentitis that led to equine abortions occurred in Japan in 2018 and 2019. EHV‐1, which is the most common pathogen causing equine infectious abortion, was not detected in any of the cases. Most inflammation lesions in our cases were extensive, spreading not only to the cervix but also to the uterine body and uterine horn (Figure 1a), although typical ascending placentitis caused by *S. zooepidemicus* or *E. coli* features inflammation of the cervical star region (LeBlanc, 2010). Most of the aborted foals weighed less than standard fetal weight, as usually found in equine abortions caused by bacteria or fungi (Murase et al., 2017). Placentitis with white–yellow exudate was found in all nine cases with placental samples (Figure 1b). Granuloma formations were found in the fetal lung, liver, spleen, and lymph nodes. Swollen lymph nodes were found in two cases: enlargement of the hilar lymph node in case 5 and the mesenteric lymph node in cases 5 and 10. Acid‐fast bacteria were detected by Ziehl–Neelsen staining in necrotic lesions of the placenta, fetal lung, lymph nodes and other organs (Figure 1c).

*Streptococcus zooepidemicus* or *E. coli* which are common pathogenic bacteria in equine abortion were not isolated from all cases except case 10. From the pathological investigations, we suspected acid‐fast bacteria to be involved in the equine placentitis and isolated acid‐fast bacteria from all cases on Middlebrook 7H11 selective agar. In all cases except case 4, necrotic lesions of the placenta had the greatest number of bacteria (data not shown), suggesting that the placenta is the most important site for bacterial isolation in mycobacterial equine abortions. All isolates were confirmed to be MAH by the PCR pattern (IS1311+, IS1245+, IS901−). In addition to MAH, *S. zooepidemicus* was isolated from the placenta and fetal stomach contents in case 10. Although this case could be a mixed infection caused by MAH and *S. zooepidemicus*, we believe that the dominant causative agent was MAH because the granuloma formations were found in the fetal organs and MAH were isolated from all the organs.

**FIGURE 2:**
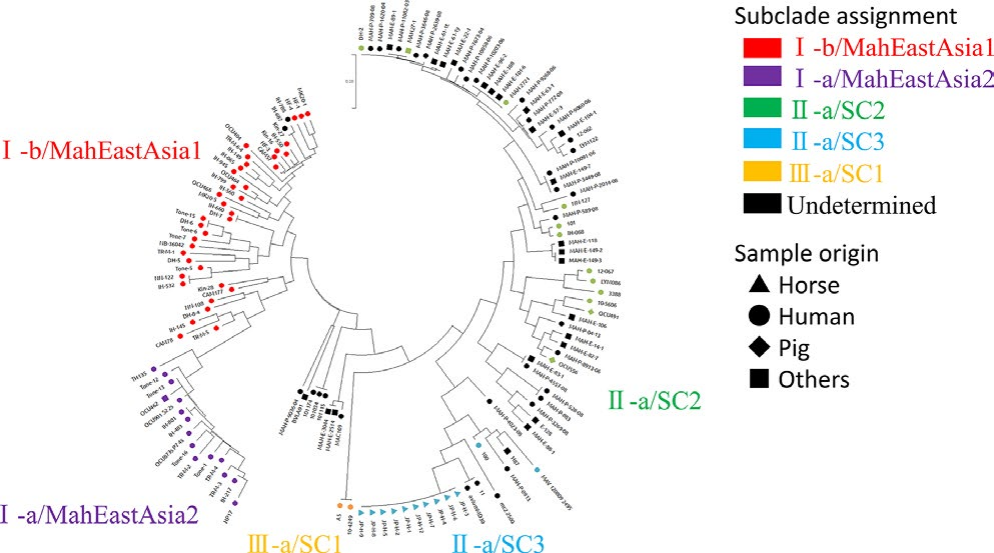
Phylogenetic tree of the 137 MAH strains. Five subclusters described in previous reports (Uchiya et al., 2017; Yano et al., 2017) were found here. Strains that were previously assigned to each subcluster and strains from this study are shown in the same colours as those of each subcluster. Sample origins are designated by the symbols shown

To determine the genetic relatedness among the 10 isolated MAH strains, we conducted VNTR and SNP analyses. Although MATR‐VNTR reportedly shows high resolution for genotyping MAH (Inagaki et al., 2009), all 10 MAH strains had the same VNTR pattern (2‐3‐3‐1‐3‐1‐3‐2‐5‐4‐2‐2‐2‐2‐2) and could not be distinguished from each other. The phylogenetic tree based on the core‐genome SNPs also placed all 10 strains together in a single group (Figure 2). These results indicate that all of the abortion cases were linked to the same infectious source. We could not find the direct contact between the mares or the bedding products which were commonly used on the farms. Therefore, further investigations of cross‐farm movements of people or veterinary medical care are needed to reveal the infectious source. Strains from East Asia form two subclusters—I‐a/MahEastAsia2 and I‐b/MahEastAsia1—and strains from Europe and the USA form other subclusters (Uchiya et al., 2017; Yano et al., 2017). All 10 strains were categorized as II‐a/SC3, being closest to human MAH strains from Taiwan (strain 11 and aviumMD30). To the best of our knowledge, these MAH strains are the first II‐a/SC3 strains isolated in Japan. One case of equine abortion from MAH infection in Hokkaido was described in 2014 (Sano et al., 2014), but we could not find any epidemiological relationship between that case and ours. Detailed comparisons based on genetic information unfortunately could not be carried out owing to a lack of information on the previous strain.

In conclusion, although horses are normally resistant to mycobacterial infections (Pavlik et al., 2004), we observed 10 mycobacterial abortions on seven horse farms in two breeding seasons. The abortions occurred during mid to late gestation, and the characteristic features of the placentitis were extensive pathological lesions and white–yellow exudates, in addition to mycobacterial granuloma formations in fetal organs. All strains had the same MATR‐VNTR pattern and were clustered together in II‐a/SC3 based on core‐genome SNPs, suggesting that the same unknown infectious source was involved in all cases. We believe that this report offers useful characteristic features of rare equine mycobacterial placentitis, and warns of the potential for future equine abortions caused by MAH.

## CONFLICT OF INTEREST

The authors declare no conflict of interest.

## AUTHOR CONTRIBUTION

**Yuta Kinoshita:** Formal analysis; Methodology; Visualization; Writing‐original draft. **Mari Takechi:** Formal analysis; Investigation; Resources; Writing‐review & editing. **Eri Uchida‐Fujii:** Writing‐review & editing. **Kunio Miyazawa:** Investigation; Writing‐review & editing. **Toshio Nukada:** Project administration; Writing‐review & editing. **Hidekazu Niwa:** Conceptualization; Writing‐review & editing.

## ETHICAL STATEMENT

The authors confirm that the ethical policies of the journal, as noted on the journal's author guidelines page, have been adhered to. All relevant guidelines for the use of animals in scientific studies were followed. The study did not include any experimentation on animals or humans, and samples were taken from natural abortion cases or routine uterine lavage.

### Peer Review

The peer review history for this article is available at https://publons.com/publon/10.1002/vms3.411.

## Data Availability

The data that support the findings of this study are available from the corresponding author upon reasonable request.
